# “Prescribing sunshine”: a national, cross-sectional survey of 1,089 New Zealand general practitioners regarding their sun exposure and vitamin D perceptions, and advice provided to patients

**DOI:** 10.1186/1471-2296-13-85

**Published:** 2012-08-17

**Authors:** Anthony Ivor Reeder, Janet Ann Jopson, Andrew Robert Gray

**Affiliations:** 1 Cancer Society Social & Behavioural Research Unit, Department of Preventive & Social Medicine, Dunedin School of Medicine, University of Otago, PO Box 913, Dunedin, 9058, New Zealand; 2 Department of Preventive & Social Medicine, Dunedin School of Medicine, University of Otago, PO Box 913, Dunedin, 9058, New Zealand

**Keywords:** Vitamin D, Skin cancer, Sun exposure, Primary care

## Abstract

**Background:**

The health effects of ultraviolet radiation vary according to wavelength, timing and pattern of exposure, personal characteristics and practices. Negative effects include skin cancers, eye diseases and immune suppression; positive effects primarily relate to endogenous vitamin D production which protects against bone disease. Drafting comprehensive guidelines regarding appropriate sun protective behaviours and vitamin D sufficiency is challenging. Advice given by general practitioners is potentially influential because they are widely respected.

**Methods:**

A survey instrument was developed, pre-tested and provided to practising GP’s, either by on-line link or mailed, reply paid hard-copy. Odds ratios, differences in means, or ratios of geometric means from regression models are reported for potential predictor variables with 95% confidence intervals.

**Results:**

Data (demographic, training, practicing, information accessing, confidence in vitamin D knowledge) suitable for analysis were obtained from 1,089 GPs (32% participation). Many (43%) were ‘not at all confident’ about their vitamin D knowledge. Recent information led 29% to recommend less sun protection during winter months and 10% less all year. Confidence was positively associated with non-‘Western’ medical training, information sources read and practising in a metropolitan centre with a medical school. Reading the Melanoma Clinical Practice Guidelines was associated with lower estimates of the amount of summer sun exposure required to obtain adequate vitamin D. Increasing years in practice was negatively associated with provision of recommended advice about summer and winter sun protection. Greater concern about vitamin D than skin cancer was expressed by females and those in practice longer.

**Conclusions:**

Concern about the potentially negative impact of skin cancer prevention on vitamin D status may undermine appropriate sun protective recommendations. Reading some educational resources was associated with confidence about vitamin D knowledge and a perception that significantly less summer sun exposure was required for those with high sun sensitivity to achieve adequate vitamin D, suggesting a potentially positive impact of such resources. Education could be targeted towards groups least likely to promote existing recommendations. Authoritative guidelines about vitamin D and sun protection would be a valued resource among GPs. Study findings are potentially valuable to help guide public policy and target interventions.

## Background

The exposure of human skin to ultraviolet radiation (UVR) can have positive and negative health effects [1]. On the positive side, the main source of vitamin D is usually endogenous synthesis from exposure of the skin to solar ultraviolet-B (UV-B) [2], although dietary intake can contribute, depending on food types, fortification and supplementation practices [3]. Vitamin D protects against rickets, osteomalacia and osteoporosis, and is positively associated with reduced risk of a number of other diseases - although convincing evidence of causality is currently lacking [4]. Apart from being the main cause of tanning in exposed skin, on the negative side, UV-B is associated with skin and lip cancers, eye diseases and immune suppression [5]. The other component of UVR, ultraviolet-A (UV-A) penetrates into deeper layers of the skin causing photo-ageing, but producing no vitamin D benefit. UV-A constitutes about 95% of ambient UVR and is present with relatively stable intensity during daylight. UV-B can vary considerably by season, time of day and location, peaking in summer around solar noon, dropping to relatively low winter levels, especially at high latitudes, and to its lowest levels early and late in the day. UV-B increases with altitude and surface reflectivity [6]. Nevertheless, solar UVR tends to be treated as a single exposure because that is what happens in everyday life, and most human evidence relates to sunlight, with wavelength effects studied almost entirely among animals using artificial sources [7].

The timing and pattern of UVR exposure has differing biological effects. Intense intermittent exposure is associated with cutaneous melanoma and basal cell carcinoma, whereas cumulative exposure is more strongly associated with squamous cell carcinoma and lip cancer [7]. Endogenous vitamin D production is most efficient during peak UVR, with less produced early and late in the day, particularly in winter. At high latitudes little vitamin D may be produced from incidental winter UVR exposure. Vitamin D production is influenced by the skin area exposed and darker coloured skin takes longer to produce a given amount of vitamin D, whereas lighter coloured skin is more susceptible to erythema.

New Zealand (NZ) represents an interesting international context within which to investigate these issues, with melanoma incidence and mortality rates among the highest in the world [8], and vitamin D ‘insufficiency’ (defined as <37.5 nmol/L) reported among 31% (22, 40) of children [9] and (defined as <50 nmol/L) 48% (45, 51) of adults [10]. A recent 2008–9 national survey found that although most NZ adults (68%) met the recommended ≥50 nmol/L level, 27% of adults fell below that level and 5% had vitamin D deficiency (<25 nmol/L), including 0.2% with severe deficiency (<12.5 nmol/L) [11].

There is on-going international debate about recommended vitamin D levels [12]. Assuming minimal sun exposure, the Institute of Medicine proposed a Recommended Dietary Allowance of 600 IU (15 μg) per day for those 1–70 years, with an upper limit of 4,000 IU (100 μg) per day for those >9 years, and a 50 nmol/L target [13], whereas the Endocrine Society Clinical Practice Guideline recommends >75 nmol/L [3]. There is also international debate about the amount of UVR exposure required to achieve a particular level of serum 25(OH)D [14,15]. which varies according to personal factors (including skin type, clothing coverage and age) as well as latitude, season and time of day.

Clear, consistent and practical public health messages are desirable, but developing such messages to achieve adequate UVR exposure for endogenous vitamin D synthesis without risking erythema is challenging. Initial NZ guidelines (summarised in Additional file 1) were developed in 2008 [16], so it is useful to know whether those recommendations are reflected in the advice that GPs provide with respect to vitamin D and UVR exposure. GPs are a respected source of health information for the general population and there is evidence, for example, that counselling related to primary care can improve sun-protective behaviours in the 10–24 year age range [17]. However, a recent Australian study identified the need for greater clarity in the advice GPs provide about sun protection and vitamin D [18], and there are similar needs internationally.

With the goal of helping to inform and guide health promotion and health education efforts, this paper:

(1)  describes the advice currently provided by GPs with respect to vitamin D sufficiency/deficiency and sun exposure/protection;

(2)  explores associations between provision of specific advice about sun exposure/protection and vitamin D and (a) demographic and practicing factors, (b) the accessing of authoritative sources of information, (c) confidence about vitamin D knowledge; all of which may help in possible intervention targeting;

(3)  identifies possible information and resource needs around vitamin D sufficiency/deficiency and sun exposure issues.

## Methods

### Study population

All NZ medical practitioners are required to register annually with the Medical Council of NZ (MCNZ) and hold a current practicing certificate. Permission to access the MCNZ register was obtained and it was accessed 1 Sept 2010. It was not possible to determine precisely how many registered practitioners were currently practising GPs, so those with ‘general practice’ as a vocational scope or any GP college noted in their qualifications were selected, cross checking with the Royal NZ College of General Practitioners (RNZCGP) 2010 membership list. The resulting ‘master file’ contained 3,450 potentially eligible practitioners.

### Survey instrument

The survey instrument (Additional file 2) drew on Australian precedent [18]. The development of that instrument included a review of content by stakeholders (‘skin cancer experts, dermatologists, vitamin D specialists, endocrinologists, behavioural scientists and members of a local general practice research group’) and testing among 20 randomly selected GPs. That survey instrument was adapted for NZ conditions and pre-tested among NZ GPs, but not further tested for validity. The NZ instrument included measures of sex and ethnicity (five category coding comprising, in order of priority, Māori, Pacific Peoples, Asian, Other, and New Zealand/European) [19], training (when, where and which qualifications received) and practising issues (years of practise, skin cancer clinic work, usual number of general practice sessions per week). The questionnaire contained items about awareness of vitamin D and its relation to sun exposure; sun protective practices; the accessing of four key information sources [16,20-22]; and perceived information needs. Several questions involved selecting items from lists, providing the potential for response bias due to list order, so items were presented in random order online and four versions of the instrument were randomly distributed in hard copy mailings. Questionnaire data were supplemented with information about whether or not the GP was based in a metropolitan area with a medical school - a potential marker of ease of access to educational opportunities. Five latitude bands were created, reflecting levels of ambient UVR, with each including at least one major population cluster.

### Data collection

An IT contractor tested the practicalities of administration using LimeSurvey version 1.87, an open source on-line survey application [23]. Once the secure survey site was activated, all recipients of the RNZCGP electronic weekly newsletter *ePulse* were notified that they could click a link and begin the survey by entering their MCNZ registration number. This link was provided for two successive weeks, Tuesday 12^th^ to Monday 25^th^ October 2010. The first survey question asked potential participants how many sessions of general practice they worked each week and only those reporting at least one were defined as currently practicing GPs and invited to complete the survey. Two weeks after the second *ePulse* mailing, a list of those remaining on the ‘master file’ who had not yet responded was provided to the MCNZ which then made direct email contact (2^nd^ November 2010, repeated 16^th^ November) with invitations and on-line links to the survey. For those not responding to these electronic opportunities, a hard copy questionnaire was posted in the first week of December 2010, with a reply paid, addressed envelope enclosed. When completing the questionnaire, participants were asked to refer to a Survey Information Sheet (Box 2) which provided contemporary definitions used in NZ regarding Fitzpatrick skin types [24], peak UVR periods and vitamin D status [25]. Ethical approval for the study was obtained through the Department of Preventive & Social Medicine and endorsed by the University of Otago Human Ethics Committee (D10/305).

### Statistical analysis

Descriptive statistics were used to summarise demographic, training and practising measures, skin cancer course completion, the accessing of skin cancer information resources and confidence in vitamin D knowledge. Where available, comparisons were made with summary data of the national medical workforce [26]. Weekday sessions were defined as either 8 am-1 pm, 1 pm-6 pm or overnight (6 pm-8 am), and weekend afternoon and overnight sessions from 1 pm-8 pm and 8 pm-8 am, respectively, following indicative locum placement terms [27]. Sessions were coded as 1–3, 4–7, and 8+ sessions per week, with the latter assumed to be equivalent to ‘full time’, allowing one day per week for administration and training. This variable was taken to indicate the ‘intensity’ of general practice work. The number of years in general practice was treated as a continuous variable. Questions asking for necessary durations of unprotected sun exposure had zeros changed to ones as reflecting more plausible responses and to allow the use of geometric means and log-transformations for regression models where appropriate. Confidence in vitamin D knowledge was collapsed into two categories, either ‘at least some confidence’ or ‘not at all confident.’ Linear and logistic regression models were used for continuous and categorical outcomes respectively. Odds ratios, differences in means, or ratios of geometric means are reported for predictor variables along with 95% confidence intervals. For linear regression models, residuals were checked for normality and homoscedasticity, with log-transformations investigated where positive skew and/or heteroscedasticity were evident and improved by the transformation. For logistic regression models, the Hosmer-Lemeshow test was used to test for lack of fit. Stata statistical software, version 12.0 was used for analyses [28]. Two-sided p < 0.05 was considered statistically significant.

## Results

### Respondent characteristics

Overall, 1,262 responded, including 63 who declined participation and 106 no longer working as GPs. These 169 were excluded, along with four others who returned questionnaires with minimal responses, leaving 1,089 for analysis, of which 686 (63%) were hard copies. We estimate this reflects a 32% participation rate, possibly underestimated due to inclusion of some on the master file who were not currently practising as GPs. The characteristics of the 1,089 participants are presented in Table 1, including comparisons with data reported for NSW GPs [18] where applicable. NZ respondents worked a median of 8 sessions per week (IQR 4 sessions) and had been practising as GPs for a mean 19.6 years (SD 10.2). Most had trained in NZ, with roughly similar numbers achieving their highest qualification before and after 2000. Respondent age was not available from the NZ electronic databases. When compared with available data on NZ medical practitioners [26], our respondents were under-representative of GPs who trained overseas (30% vs 42%), and over-representative of women (51% vs 44%). 

**Table 1:** **Characteristics of the study participants and comparison with findings reported for NSW GPs**[18], **where applicable**

	**NZ*n***	**NZ*%***	***NSW %***
**Gender**			
Male	533	49.0	*52.1*
Female	555	51.0	*47.9*
*Missing data*	*1*		
**Ethnicity* (multiple identification possible) ***			*N/A*
Māori	22	2.0	*-*
Pacific	2	0.2	*-*
Asian	134	12.4	*-*
NZ European/European	933	86.4	*-*
Other	15	1.4	*-*
*Missing data*	*9*		
**Location**			*N/A*
Metropolitan centres with a medical school	547	50.2	*-*
All other	542	49.8	*-*
*Missing data*	*0*		
**Latitude bands for location of practice**			*N/A*
Upper N: 34 to 36.59°	344	31.8	*-*
Mid-N: 37 to 39.59°	282	26.0	*-*
Lower N/upper S: 40 to 41.59°	199	18.4	*-*
Mid-S: 42 to 44.59°	171	15.8	*-*
Lower S: 45 to 47°	87	8.0	*-*
**GP practice (years)**			
< 5	94	8.7	*5.0*
5 to 10	159	14.7	*9.8*
11 to 20	324	29.9	*25.9*
> 20	505	46.7	*59.3*
*Missing data*	*7*		
**Practice sessions per week**			
1 to 3	117	11.1	*-*
4 to 7	388	36.7	*-*
≥ 8 (‘full time’)	553	52.3	*81.5
*Missing data*	*31*		
**Place of medical graduation**			
NZ	767	70.4	*-*
US/UK/other European	191	17.6	*-*
SE Asian	30	2.8	*-*
S Africa	39	3.6	*-*
All other	31	2.9	*-*
Australia	28	2.6	*72.9*
*Missing data*	*3*		
**Highest medical qualification**			
Medical degree	173	15.9	*33.5*
Graduate certificate/diploma	76	7.0	*11.1*
Master’s degree	21	1.9	*4.0*
College fellowship	799	73.4	*46.4*
Research doctorate	16	1.5	*1.0*
Other	3	0.3	*4.0*
*Missing data*	*1*		
**Year received highest medical qualification**			
Before 1980	88	8.2	*30.4*
1980-1999	478	44.7	*44.8*
2000 and after	504	47.1	*24.9*
*Missing data*	*19*		
**Skin cancer course completion**			
Yes	190	17.4	*10.1*
**Confidence about vitamin D knowledge**			
Very confident	34	3.2	*13.5*
Confident	583	54.0	*77.3*
Not at all confident	462	42.8	*9.2*
*Missing data*	*10*		
**Information sources read* (multiple responses possible in NZ survey)) ***			
CSNZ (Cancer Council Australia)	219	20.1	*20.0*
WHO	51	4.7	*-*
Clinical Practice Guidelines (melanoma)	476	43.7	*-*
NHMRC NMSC guidelines	123	11.3	*-*

* The respective use of the ‘full time’ descriptor may not be strictly comparable.

### Reading of information sources and other plausible predictors of confidence about vitamin D knowledge

From a list of four authoritative sources regarding sun exposure, skin cancer and vitamin D, respondents were asked to indicate which they had read (Table 1). Most commonly read was the Clinical Practice Guidelines for the Management of Melanoma [20], followed by the CSNZ position statement on the risks and benefits of sun exposure [16], the Australian treatment and management guidelines for non-melanoma skin cancer [21], and the IARC report on Vitamin D and Cancer [22]. Overall, 590 (54.9%) of the 1,075 respondents with valid data had read at least one of these documents.

Plausible statistical predictors of GP confidence about their vitamin D knowledge were investigated (Table 2). In multivariable analysis, confidence about vitamin D knowledge was significantly and positively associated with having received training in SE Asia or an ‘other’ location; having read the CSNZ or WHO/IARC source documents; practising in a major metropolitan centre with a medical school, and residence in all latitude bands except the upper North compared to the Lower North Island/Upper South Island.

**Table 2:** Odds ratios (OR) and confidence intervals (CI) for association of factors with GP confidence about their vitamin D knowledge

	**Unadjusted**				Adjusted^*^			
	***OR***	***95 % CI***		***p***	***OR***	***95 % CI***		***p***
		**Lower**	**Upper**			**Lower**	**Upper**	
**Location*** Ref. all other sites *				0.001				0.001
Major metropolitan centre with a medical school	1.49	1.17	1.90		1.75	1.27	2.41	
**Place of medical graduation**				<0.001				<0.001
US/UK/Other Euro	0.73	0.53	1.00		0.81	0.58	1.14	
SE Asian	6.75	2.03	22.42		5.65	1.64	19.53	
South African	0.67	0.35	1.29		0.84	0.42	1.67	
All others	4.87	1.68	14.09		5.43	1.80	16.38	
**Female***Ref. male*	0.80	0.63	1.02	0.077	0.76	0.56	1.03	0.079
**Years as a GP*** (per 5 years) *	1.06	1.00	1.12	0.061	1.02	0.95	1.09	0.541
**Latitude Band** of practice * Ref. Upper-South (40–41.59*°)				0.002				0.026
Upper-North (34–36.59°)	1.95	1.37	2.78		1.43	0.96	2.11	
Mid-North (37–39.59°)	1.63	1.13	2.35		1.81	1.20	2.75	
Mid-South (42–44.59°)	1.81	1.20	2.74		1.79	1.15	2.81	
Lower-South (45-47°)	2.27	1.34	3.83		1.89	1.08	3.32	
**Information sources read**								
CSNZ	2.82	2.01	3.95	<0.001	2.33	1.62	3.35	<0.001
WHO/IARC	5.98	2.53	14.14	<0.001	3.59	1.46	8.86	0.006
Clinical Practice Guidelines	1.38	1.08	1.76	0.010	1.24	0.94	1.64	0.129
NHMRC	1.34	0.91	1.98	0.139	0.92	0.59	1.44	0.709
Any of the above	1.61	1.26	2.06	<0.001				
**Number of sessions*** Ref ≥8 *				0.172				0.188
1-3	1.41	0.93	2.13		1.51	0.96	2.35	
4-7	1.47	0.98	2.19		1.31	0.83	2.07	
**Skin cancer training course**				0.006				0.097
Completed course	1.59	1.14	2.22		1.36	0.95	1.95	

^*^ Adjusted for all other variables listed in the table.

### Estimated summer sun exposure times to achieve adequate vitamin D

Respondents were asked: “How many minutes of unprotected sun exposure of (the) face, hands and arms is necessary just after 9 am in summer in your region for a person with *HIGH sun sensitivity* (Fitzpatrick skin types I and II – see Additional file 3) to get adequate vitamin D?” Another question sought similar information for those with low sun sensitivity (Fitzpatrick skin types V and VI). Reported exposure times ranged from 1 to 240 minutes (geometric mean 14.8, geometric SD 1.8) and 1 to 300 minutes (geometric mean 26.7, geometric SD 2.0) for those with high and low sensitivity, respectively. The associations between these perceptions and potential predictors (those listed in Table 2) were investigated (see Additional file 4). Having read the Melanoma Clinical Practice Guidelines was the factor most strongly associated with lower exposure time (i.e. more protective against skin cancer) estimates (ratio of means 0.88, 0.82-0.95, p = 0.001), whereas years in practice (ratio of means 1.02 per five years, 1.00-1.04, p = 0.026) and having read the CSNZ position statement (ratio of means 0.90, 0.82-0.99, p = 0.026) were weakly positively associated. The inclusion of ‘confidence’ in the model did not change these findings.

When the same factors were investigated in relation to responses to a similar question about people with low sun sensitivity, a training location other than Australia and NZ (overall p = 0.014) was only statistically significant different for ‘all others’ (ratio of means 0.69, 0.53-0.89, p = 0.004), and having read the clinical practice guidelines (0.90, 0.83-0.99, p = 0.026) was weakly negatively associated with lower exposure estimates (i.e. estimates more risky for vitamin D deficiency) in the multivariable model. Having read the WHO/IARC resource (1.25, 1.02-1.54, p = 0.033) was weakly positively associated with higher estimates.

### Sun protection/exposure advice

Participants were asked, regarding summer and winter, separately: “As a result of your awareness of vitamin D, what sun protection advice do you generally give your patients?” For summer, most GPs advised patients ‘to use sun protection at all times during peak UV’ (statement 1) (Table 3). We treated endorsement of either statement 1 or a combination of statements 1 and 3 as most congruent with current recommendations. Overall, this ‘correct’ advice was provided by 71% (n = 766) of the 1074 with usable data. We also examined in multivariable analysis which, if any, factors (those listed in Table 2) were associated with provision of ‘correct’ summer advice. There were differences in training location (overall p = 0.014) but apart from receiving training in the US/UK/Europe rather than Australia or NZ (OR 1.54, 1.04-2.29, p = 0.032), the number of years practicing as a GP was the only significantly associated factor, with increasing years in practice negatively associated with provision of such advice – a 0.86 OR for every five years of practice (0.80 to 0.93, p < 0.001) (Additional File 3).

**Table 3:** **Sun protection advice provided, by season, with comparisons between NZ and NSW**[18]**GPs**

**Sun protection advice provided to general population**	**Summer**		**Winter**		**Generic**	
	**NZ*%***	***NSW %***	**NZ*%***	***NSW%***	**NZ%**	***NSW%***
**Statement**						
1.To use sun protection at all times during peak UV	70	*55*	21	*33*	-	*-*
2. To use sun protection most of the time during peak UV, but to receive some direct sunlight during that time	17	*26*	30	*33*	-	*-*
3. Not to use sun protection outside of peak UV times and receive direct sunlight during that time	11	*15*	36	*27*	-	*-*
4. Not to use sun protection at any time	2	*	13	*2*	-	*-*
**Has the vitamin D advice you have received in the past 12 months influenced the sun protection advice you now provide?**						
Now recommend less sun protection	2	*	29	*20*	10	*
No change in sun protection advice	-	*-*	-	*-*	59	*68*

* Not reported.

For winter, the most common advice was ‘not to use sun protection outside of peak UV times and receive direct sunlight during this time’ (Table 3). As for summer, a similar model was constructed with respect to winter advice. Statement options 1, 3 or a combination of 1 and 3 were treated as most congruent with current winter advice. Overall, that advice was provided by 54% of the 1073 with usable data. No statistically significant associations were found or any changes in the multivariable model when ‘confidence’ was added. For those patients ‘at increased risk of vitamin D deficiency’, recommendation 4 was more commonly selected than for the general population in winter (24% vs 13%), otherwise the winter advice provided was not markedly different.

When asked ‘How much information about vitamin D have you received in the last 12 months?’ most (45%) indicated ‘more than usual’, 38% ‘about the same as usual’ and 4% ‘less than usual.’ The remainder indicated that none was received. When asked ‘Has the information you have received about vitamin D in the last 12 months influenced the sun protection advice you now provide to your patients?’ most indicated that there had been no change (Table 3).

### Responses to the statement matrix, including expression of greater concern about vitamin D than skin cancer

A matrix of eight statements was provided for respondents to indicate either agreement or disagreement with each. The results (Table 4) are ranked by percentage of ‘agreement’ (i.e. ‘strongly agree’ and ‘agree’ responses combined). Analysing the dichotomised results (i.e. the four response categories collapsed into either ‘agree’ or ‘disagree’) in the multivariable context of those factors identified in Table 2, significantly higher odds of agreement with statement 2 were found for those trained in S.E. Asia (OR 3.9, 1.4-10.5, overall p = 0.006) or resident in a city with a medical school (OR 1.5, 1.12-2.09, p = 0.008). The addition to the model of confidence about vitamin D knowledge did not markedly change either the direction or strength of these findings. With respect to statement 3, being female (OR 1.6, 1.1-2.4, p = 0.014) and more years in practice (OR 1.1 per five years, 1.0-1.2, p = 0.028) were the only factors significantly associated with agreement. With the addition of confidence, that variable was significant (OR 1.4, 1.0-2.0, p = 0.039), but did not markedly alter the other results. In similar analyses for Statement 8, the only significant difference identified was that females were more likely than males to agree (OR 1.8, 1.3-2.5, p < 0.001). With the addition of confidence, the only marked change was that higher confidence was associated with being less likely to agree (OR 0.65, 0.49-0.86, p = 0.003).

**Table 4:** **Percentages of GPs indicating agreement**^**1**^**with statements and comparison with findings reported for NSW GPs**[18].

**Statement**	**Agree**	
	**NZ*%***	***NSW %***
1. Clinical guidelines regarding vitamin D deficiency would be useful	97	*97*
2. I am concerned that my patients may not be getting enough vitamin D	87	*83*
3. Skin cancer prevention messages contribute to the development of vitamin D deficiency	81	*68*
4. Vitamin D reduces the risk of cancer	68	*53*
5. My patients need to spend more time in the sun to get enough vitamin D to be healthy	58	*60*
6. Information about vitamin D is not readily available for GPs	50	*53*
7. The vitamin D status of my patients influences the sun protection advice I provide	45	*65*
8. It is more important to stay out of the sun than get enough vitamin D	35	*32*

^**1**^ Percentages of respondents selecting ‘strongly agree’ or ‘agree’ combined and ranked by NZ response frequency.

## Discussion

The results of our nationwide study generally reinforce the findings of the NSW statewide survey [18], but further studies in other countries would be useful to assess the need for guidelines and their evaluation. We were able to substantially extend the NSW findings by including in our analyses consideration of potential latitude band effects and investigation of plausible statistical predictors of confidence about vitamin D knowledge and the provision of recommended advice about sun protection and vitamin D, both for the general population and those at increased risk of skin cancer or vitamin D deficiency. We also investigated statistical predictors of agreement/disagreement with a range of statements relating to perceptions regarding vitamin D and skin cancer.

### Reading of specific information sources and confidence about vitamin D knowledge

Overall, 20% of GPs reported having read the CSNZ position statement [16], similar to the 24% in NSW who had read comparable Australian guidelines [18]. However, our finding that almost five times more NZ than NSW GPs reported being ‘not at all confident’ about their vitamin D knowledge indicates that lack of confidence is more pressing in NZ. Practising in a major metropolitan centre with a medical school was positively associated with confidence, giving support to the hypothesis that such locations may provide better access to educational opportunities. Both NZ and NSW GP’s almost unanimously agreed that clear clinical guidelines about vitamin D ‘would be useful’, providing a very clear indication of what they wanted. A NZ Consensus Statement on Vitamin D and Sun Exposure has since been published [29], in part, as a response to preliminary findings of the present study, thereby providing future opportunities to assess whether or not such a resource is associated with change in confidence and the advice provided.

### Estimated summer sun exposure times required to achieve adequate vitamin D

For patients with high sun sensitivity (defined as Fitzpatrick Skin Types I & II), the mean summer sun exposure time of the unprotected face, hands and arms that GPs perceived would be required (before 10 am, after which hour current NZ guidelines recommended routine sun protection when the UVI is ≥ 3) in order to obtain adequate vitamin D was approximately 15 minutes. Assuming that the UVI was no higher than 3, sufficient vitamin D should be able to be produced in that period while erythema could be avoided [30]. Such a mean exposure time may, broadly, be considered compatible with the recommendation of ‘a few minutes of sunlight on either side of the peak UVR periods’ [16]. For those with low sun sensitivity (defined as Skin Types V-VI) the perceived mean time was considerably longer (27 minutes), consistent with the longer exposure time required for darker skin types. However, for both skin type groups the reported range was wide, with some GPs providing estimates of 2 hours and 4 hours as appropriate for those of low and high sun sensitivity, respectively. This should be of concern and more conservative estimates should be a target for information strategies to achieve. It was not possible to compare these NZ estimates with those reported for NSW as in that study the estimated period of exposure was during peak UVR [18], a behaviour incompatible with existing NZ recommendations not to seek ‘deliberate exposure at peak UVR times’ [16]. Even so, 22% of Australian GPs were reported as believing 30 minutes during peak UVR would be required for a person of average sun sensitivity to achieve adequate vitamin D, whereas the Australian guidelines indicate that only 6 to 8 minutes would be required at 10 am in Sydney during summer. While acknowledging the challenges, we agree that there is ‘a need for an easier and quicker way for doctors to calculate safe UV exposure and for determining risk status to help them provide tailored advice’, such as ‘desktop decision aids, with computer algorithms that take into account the complexities of skin type, weather and location’ [18].

### Sun protection/exposure advice

Most NZ GPs (70%) and more than in NSW (55%) advised the currently recommended summer sun protection strategy (‘to use sun protection at all times during peak UV’), although 17% (NSW 26%) recommended sun protection ‘most of the time during peak UV, but to receive some direct sunlight during that time.’ Winter advice was less restrictive, with 36% (NSW 33%) advising patients ‘not to use sun protection outside of peak UV times and receive direct sunlight during this time’, and 13% (2% in NSW) advising patients ‘not to use any sun protection at any time’ during winter. For patients ‘at increased risk of vitamin D deficiency’ the latter advice was more commonly provided than for the general NZ population (24% vs 13%), otherwise the winter advice provided by NZ GPs to these groups did not differ markedly.

When asked ‘How much information about vitamin D have you received in the last 12 months?’ almost the same percentages in NZ (45%) as NSW (46%) indicated ‘more than usual.’ This was despite the surveys having been conducted in different years (NSW: Aug-Dec. 2009; NZ: Oct-Nov. 2010), indicating virtually no difference in perceptions of the balance of information available to GP’s during the two time periods in the two geographical areas. Most NZ GP’s (59%) had not changed their advice as a result of information received during the past 12 months, which was consistent with, but less stable than found in NSW (68%). We found a somewhat stronger shift in NZ than NSW towards recommending less winter protection (29% vs 20%) and, furthermore, 10% reported a shift towards recommending less protection all year round (a response option not reported for NSW).

### Reading of information sources and other factors associated with confidence about vitamin D knowledge and ‘quality’ of advice

We investigated plausible statistical predictors of (1) confidence about vitamin D knowledge and (2) the provision of ‘correct’ advice. GPs who trained outside NZ/Australian/other ‘western’ centres were more confident about their vitamin D knowledge, but less likely to advise routine sun protection at times of high summer UVR. This finding is consistent with possibly less awareness about the seasonally extreme UVR levels in NZ, which can be almost 50% higher than at comparable northern hemisphere latitudes in summer [31]. The provision of specific information about this significant difference may, therefore, be of value during on-going clinical education. However, among all participants, completion of a skin cancer training course was associated neither with confidence nor provision of ‘quality’ advice. GPs in practice longer were also less likely to advocate sun protection at times of high UVR in summer months, consistent with possibly increasingly emphasis on protective strategies during recent medical training regimes. This would seem to reinforce the need for training updates in skin cancer prevention. When we examined latitude gradient of Medical Council Register address against confidence in knowledge about vitamin D, the lowest confidence levels were in the 40-42°S latitude band which includes the capital city, Wellington (North Island) and the Nelson region (northern South Island). There seems no clear explanation for this association with proximity to the national political capital. In a multivariable context, although the reading of some specific information sources was positively associated with confidence and lower perceived exposure times for sufficient vitamin D in summer among those with high sun sensitivity, there was no association with the categorical ‘correctness’ of advice provided. These findings provide partial confirmation of the potential value of such resources.

### Responses to statement matrix and expression of greater concern about vitamin D than skin cancer

Responses to the matrix of statements (Table 4) indicate widespread concerns about vitamin D deficiency and the potentially negative impact of skin cancer prevention messages on vitamin D status. A very similar pattern was found in NSW, although more NZ (81%) than NSW (68%) GPs indicated agreement with the statement that ‘skin cancer prevention messages’ ‘contribute to the development of vitamin D deficiency.’ Bonevski et al. [18] found that females were more likely to express such concerns and we confirmed this among NZ GPs for two of the three related questionnaire items. However, we also found evidence of statistically significant associations with having been trained in S.E. Asia, residence in a city with a medical school, being in practice for a greater number of years and expressing greater confidence about vitamin D knowledge. Consideration should be given to each of these factors when targeting educational interventions.

The potential for these perceptions to undermine appropriate sun protection messages in the context of sometimes extreme NZ summer UVR levels should be of concern. As Bonevski et al. note, ‘although vitamin D plays an important role in bone health, the evidence regarding the other health benefits of vitamin D remains inconclusive’ [18] and this situation continues [22][13]. Nevertheless, to pre-empt perceptions of division within the scientific community and inconsistency in public health messages, it remains important for sun protection messages to take into account vitamin D issues, in particular, known lower vitamin D levels in NZ associated with living at high latitude, non-European ethnicity and more highly pigmented skin [32]. This may be challenging, but follows a predicted pattern of the need to develop more targeted messages for specific population groups [33], and reinforces the call for decision aids [18].

### Study strengths and limitations

Our procedures meant that we were unable to employ all of the recommended strategies for improving response rates in surveys of physicians, in particular, the use of financial incentives [34]. However, our national survey drew on the two most relevant professional organisations (RNZCGP and MCNZ) to provide contact with GPs, and the participation rate in our study was 32%, slightly higher than obtained for the comparable study in the Australian state of New South Wales which used financial incentives [18]. Those authors argue that their reported level of response was comparable to other practitioner surveys and the literature suggests that there may be only a weak association between response rates and bias, if any [35]. Participants in our survey differed from the national population of practitioners in terms of higher participation by females than males and part-time than full time GPs, similar to the Australian findings [18]. Other demographic data were not accessible to permit valid comparisons between respondents and the NZ practitioner population. These factors should be taken into account when extrapolating to the GP population. There were similarities and differences between the NZ national and NSW state samples. Somewhat fewer in NZ had been practising for longer than 20 years, in both cases most had graduated within the country of survey, but many more in NZ than Australia reported a college fellowship as their highest medical qualification, more had received their highest medical qualification since 2000 and many fewer before 1980; 17% had completed a skin cancer course whereas only 9% of the NSW sample had either enrolled in or completed such a course. Many fewer NZ GPs practiced fulltime, although the definition of full time employment differed, in NZ being based on the reported number of sessions worked, but dichotomously self-reported in Australia.

The results of our study add substantially to knowledge, not only largely confirming the findings of similar prior NSW research, but also extending that research by investigating, in a multivariable context, factors associated with GPs’ perceptions and the advice they provide. These multivariable analyses allowed us to identify some significant differences in perceptions and advice according to gender, location of medical graduation, number of years in practice, confidence about vitamin D knowledge, residence in a city with a medical school and information sources read. Among the other strengths of our study was the inclusion of randomisation in the order of presentation of lists of response options, something which was not reported for the NSW survey, but which adds confidence to the findings. The nation-wide reach of our study also permitted investigation of potential latitudinal differences in perceptions and advice. That none were found indicates homogeneity, although there is justification for some variation, given significant regional differences in seasonal UVR levels.

## Conclusions

The widespread concern expressed about vitamin D deficiency and the potentially negative impact of skin cancer prevention messages on vitamin D status, both confirms and strengthens a similar NSW finding and needs to be addressed. The potential for these perceptions to undermine appropriate sun protection messages, particularly in the context of the potentially extreme UVR levels of a NZ summer, should be of concern. Completion of a skin cancer training course was not associated with the quality of GP’s sun protection and vitamin D advice, nor confidence about vitamin D knowledge, so the content of such courses may benefit from re-examination in the context of its broader relevance and impact. However, the reading of some (or any) of the four identified educational resources was associated both with confidence about vitamin D knowledge and a perception that significantly less summer sun exposure was required for those with high sun sensitivity to achieve adequate vitamin D. Although no associations were found with the categorical ‘quality’ of advice, these findings suggest such resources can have a positive impact. Given their less protective responses, consideration should be given to targeting educational interventions towards those who have been in practice for a greater number of years or received medical training in S.E. Asia.

As a result of our research it became clear that clinical guidelines about vitamin D and sun protection would be a valued resource among GPs, and it is encouraging that the Ministry of Health has recently released a Consensus Statement in collaboration with the CSNZ [29], in part, as a response to advice about our preliminary findings. Making such a document widely available from an authoritative source may go some way towards countering unbalanced reports and overcoming the dilemmas that GPs face. The development of desktop computer aids, as suggested by Bonevski et al., [18] would seem to offer a promising approach to helping identify those at greatest risk of harm from either excess UVR exposure or insufficient vitamin D.

Although confirmation of our findings in other studies is desirable, internationally, the results highlight the value of conducting such a survey, particularly when that ‘baseline’ is followed by the development of a consensus statement, thereby permitting possible evaluation of impact on GPs practises.

## Competing interests

AIR participated in the Consensus Statement Workshop process which provided advice for the drafting of the Ministry of Health and Cancer Society of New Zealand: *Consensus Statement on Vitamin D and Sun Exposure in New Zealand*[29].

## Authors’ contributions

AIR initiated and supervised the study, was primarily responsible for writing successive drafts of the paper and submitting the final version. JAJ was appointed to work on study development and data collection; wrote an initial descriptive report of the results and commented on subsequent drafts. ARG advised on statistical issues, performed the statistical analyses and contributed to successive drafts of the paper. AIR & JAJ received funding from the CSNZ and the University of Otago. ARG was employed by the University of Otago. All authors read and approved the final manuscript.

## Authors’ information

AIR is Associate Professor and Director of the Cancer Society of NZ Social & Behavioural Research Unit.

## Pre-publication history

The pre-publication history for this paper can be accessed here:

http://www.biomedcentral.com/1471-2296/13/85/prepub

## Supplementary Material

Additional file 1 Summary of key New Zealand guidelines for reducing risk of vitamin D deficiency and skin cancers. Click here for file

Additional file 2 Questionnaire instrument. Click here for file

Additional file 3 Survey information sheet provided to all respondents. Click here for file

Additional file 4 Analysis of other associations. Click here for file
