# Supplementary material for: “Prescribing sunshine”: a national, cross-sectional survey of 1,089 New Zealand general practitioners regarding their sun exposure and vitamin D perceptions, and advice provided to patients
Source: BMC Fam Pract. 2012 Aug 17;13:85. doi: 10.1186/1471-2296-13-85 (PMC3460728; doi:10.1186/1471-2296-13-85)
Supplement: Additional file 1 — Summary of key New Zealand guidelines for reducing risk of vitamin D deficiency and skin cancers. [file 1471-2296-13-85-S1.pdf]

Additional File 1. Summary of key New Zealand guidelines for reducing risk of vitamin D deficiency and skin cancers.[a]

1. General population

September to March (August to April in the north) - higher UVR period: use sun protection[b] when the UVI is ≥3, paying special attention to the period between 11am and 4pm.

Sufficient vitamin D (50nmol/L) should be able to be obtained from typical outdoor activities outside peak UVR times.

April to August - lower UVR period: some sun exposure when the UVI is below 3 may help to increase vitamin D status without increasing the risk of skin damage.

At high altitudes or near highly reflective surfaces (e.g. snow or water): sun protection should be used throughout the year.

2. People at increased risk of skin cancer

For Fitzpatrick skin types I and II,[b] exposing the face, arms and hands or the equivalent area of skin to a few minutes of sunlight on either side of the peak UVR periods most days of the week should provide sufficient vitamin D in summer.

People with a history of sun damage and skin cancer, or organ transplant recipients and those taking medicines that affect photosensitivity should use sun protection all year round.

3. People at increased risk of vitamin D deficiency[b]

People with naturally dark skin (Fitzpatrick skin types V and VI)[b] should be aware of the need for protection at high UVR times, may not need to wear sunscreen, but should wear a hat because eye damage affects all skin types.

Those at high risk, using sun protection year round, or with full body clothing coverage for religious or cultural reasons and vitamin D deficient mothers of breastfed babies, should discuss their vitamin D requirements with their health practitioner.

[a] Position Statement. The Risks and Benefits of Sun Exposure in New Zealand. Cancer Society of New Zealand, 2008.

[b] As defined in **Additional File 2.**
