# Supplementary material for: “Prescribing sunshine”: a national, cross-sectional survey of 1,089 New Zealand general practitioners regarding their sun exposure and vitamin D perceptions, and advice provided to patients
Source: BMC Fam Pract. 2012 Aug 17;13:85. doi: 10.1186/1471-2296-13-85 (PMC3460728; doi:10.1186/1471-2296-13-85)
Supplement: Additional file 2 — Questionnaire instrument. [file 1471-2296-13-85-S2.pdf]

Questions 1 to 9 are about you and your practice.

1. **Please enter your Medical Council Registration Number** ☐☐☐☐☐

2. **How many sessions do you usually work in General Practice per week?**

☐☐ **sessions**

This survey is designed to be completed by those currently practising as General Practitioners in NZ. If you are not currently practising, please return the uncompleted survey to us in the enclosed envelope.

3. **Are you male or female?**

Please tick the appropriate box

Male ☐1
Female ☐2

4. **Which ethnic group(s) do you belong to?**

Please tick any that apply to you:

Maori ☐1
Pacific ☐2
Asian ☐3
New Zealand European ☐4
Other ☐5 e.g. Dutch, Japanese, Tokelauan
Please specify ________________________________

5. **For how many years have you been practising as a GP?** ☐☐ **years**

6. **Which of the following would you say is your <u>highest</u> medical qualification?**

Please tick ONE box only:

Medical degree ☐1 (MB BS, MB Ch, BMed etc)
Graduate certificate ☐2
Graduate diploma ☐3
Coursework Master's degree ☐4
Research Master's degree ☐5
College fellowship or equivalent ☐6 (FRACGP, MRCGP, FACRRM etc)
Research doctorate ☐7 (PhD, MD, etc)
Other ☐8 ***Please specify*** ________________________________

7. **Which year did you receive your highest medical qualification? *(YYYY)***

☐☐☐☐

8. In which country or countries did you train in medicine?

Please tick as many as required:

Australia ☐[1]
United States of America ☐[2]
United Kingdom ☐[3]
New Zealand ☐[4]
Other ☐[5] ***Please specify*** _______________________________

9. Have you completed or are you enrolled in special training in <u>skin cancer</u>?

No ☐

	**Completed**	**Enrolled**
Short course	☐	☐
Graduate Certificate	☐	☐
Diploma	☐	☐
Masters	☐	☐
Other	☐	☐

Please specify _________________________

10. Do you work in a skin cancer clinic part-time?

Please tick ONE box only:

Yes ☐
No ☐

The next questions are about your awareness of vitamin D, its relationship to sun exposure, your sources of information and information needs. We do not expect you to consult literature or notes. Please answer according to your current understanding and beliefs.

11. Which of the following do you believe may <u>prevent</u> vitamin D deficiency in the general population? ***Please tick as many as required:***

Daily exposure to sunlight outdoors at peak UV times of the day ☐[01]
A course of high-dose (50,000 IU) vitamin D supplements ☐[02]
Daily exposure to sunlight outdoors at low UV times of the day ☐[03]
Relaxation of sun protection during winter ☐[04]
Daily exposure to artificial UV light ☐[05]
There is no effective way to prevent vitamin D deficiency ☐[06]
Daily calcium supplements ☐[07]
Relaxation of sun protection throughout the year ☐[08]
Adequate intake of vitamin D fortified foods ☐[09]
Adequate physical activity ☐[10]
Daily low dose (2,000 IU or less) vitamin D supplements ☐[11]

12. Which is the <u>main</u> source of vitamin D in New Zealand?

Please tick ONE response in each column

	**Summer**	**Winter**
Fortified cereals	☐[1]	☐[1]
Exercise	☐[2]	☐[2]
Fish with a high fat content (e.g. sardines, wild salmon)	☐[3]	☐[3]
Fortified milk products and margarine	☐[4]	☐[4]
Exposure to sunlight outdoors	☐[5]	☐[5]
Supplements	☐[6]	☐[6]
Not sure	☐[7]	☐[7]

Exposure to artificial UV light ☐8 ☐8

13. How many minutes of unprotected sun exposure of face, hands and arms is necessary just after 9am in summer in your region for a person with *HIGH sun sensitivity (Fitzpatrick Skin Types I-II)* to get adequate vitamin D?

minutes

14. How many minutes of unprotected sun exposure of face, hands and arms is necessary just after 9am in summer in your region for a person with *LOW sun sensitivity (Fitzpatrick Skin Types V-VI)* to get adequate vitamin D?

minutes

15. Which are the more common vitamin D related patient enquiries you receive?

Please tick as many as required:

Not applicable – do not receive any patient enquiries regarding vitamin D ☐1
Advice regarding how much time they should spend out in the sun ☐2
Information about vitamin D following media reports ☐3
Advice regarding the use of sun protection and effects on vitamin D ☐4
Information about sources of vitamin D ☐5
Requests for prescribed vitamin D ☐6
Requests for complementary and alternative therapies ☐7
Requests for serum vitamin D level tests ☐8

16. How confident do you feel about your vitamin D knowledge?

Please tick ONE box only:

Very confident ☐1
Confident ☐2
Not at all confident ☐3

17. How have you mostly obtained information about vitamin D deficiency?

Please tick ONE box only:

Journals ☐01
Courses/training programs ☐02
Never obtained information about vitamin D ☐03
Industry literature and promotions ☐04
Colleagues ☐05
Mass media ☐06
Professional guidelines ☐07
Patients ☐08
Online/internet (non-professional sources) ☐09
Medical training ☐10

18. Have you read any of the following?

Please tick ONE for each document:

	Yes	No
The Risks and Benefits of Sun Exposure in New Zealand. Cancer Society of New Zealand. 2008	☐	☐
WHO/IARC. Vitamin D and Cancer. 2008	☐	☐
Clinical Practice Guidelines for the Management of Melanoma in Australia & NZ. 2009	☐	☐
NHMRC Clinical Practice Guidelines: Non melanoma skin cancer. 2003	☐	☐

19. As a result of your awareness of vitamin D, what sun protection advice do you generally give your patients during <u>SUMMER</u>? ***Please tick ONE box only:***

To use sun protection at all times during peak UV	☐1
To use sun protection most of the time during peak UV, but to receive some direct sunlight during that time	☐2
Not to use sun protection outside of peak UV times and receive direct sunlight during this time	☐3
Not to use sun protection at any time during the day	☐4

20. As a result of your awareness of vitamin D, what sun protection advice do you generally give your patients during <u>WINTER</u>? ***Please tick ONE box only:***

To use sun protection at all times during peak UV	☐1
To use sun protection most of the time during peak UV, but to receive some direct sunlight during that time	☐2
Not to use sun protection outside of peak UV times and receive direct sunlight during this time	☐3
Not to use sun protection at any time during the day	☐4

21. As a result of your awarentess of Vitamin D, what sun protection advice do you give patients who are at <u>INCREASED risk</u> of vitamin D deficiency during <u>WINTER</u>? ***Please tick ONE box only:***

To use sun protection at all times during peak UV	☐1
To use sun protection most of the time during peak UV, but to receive some direct sunlight during that time	☐2
Not to use sun protection outside of peak UV times and receive direct sunlight during this time	☐3
Not to use sun protection at any time during the day	☐4

22. How much information about vitamin D have you received in the last 12 months?

Please tick ONE box only:

More than usual ☐1
Less than usual ☐2
About the same as usual ☐3
None ☐4

23. Has the information you have received about vitamin D in the last 12 months influenced the sun protection advice you now provide to your patients? ***Please tick ONE box only:***

Yes, I now recommend less sun protection during summer ☐1
Yes, I now recommend less sun protection during winter ☐2
Yes, I now recommend less sun protection all year round ☐3
No changes to my sun protection advice ☐4

24. To what extent do you agree or disagree with each of the following statements?

Please circle ONE option for each statement:

	**Strongly agree**	**Agree**	**Disagree**	**Strongly disagree**
It is more important to stay out of the sun than get enough vitamin D.	SA	A	D	SD
Vitamin D reduces the risk of cancer	SA	A	D	SD
Clinical guidelines regarding vitamin D deficiency would be useful.	SA	A	D	SD
Skin cancer prevention messages to cover up in the sun contribute to the development of vitamin D deficiency.	SA	A	D	SD
My patients need to spend more time in the sun to get enough vitamin D to be healthy.	SA	A	D	SD
The vitamin D status of my patients influences the sun protection advice I provide.	SA	A	D	SD
Information about vitamin D is not readily available for GPs.	SA	A	D	SD
I am concerned that my patients may not be getting enough vitamin D.	SA	A	D	SD

This last section is about your patients. We understand that it may be difficult to give exact answers. We do not expect you to check your records or notes, but would appreciate your best estimate.

25. Please estimate how many patient contacts you have <u>IN AN AVERAGE WEEK</u>?

☐☐☐ **patients**

26. Please estimate the number of patients you have seen for skin cancer (including melanoma) and related effects in the <u>LAST 12 MONTHS</u>?

treated ☐☐☐ **patients**

referred ☐☐☐ **patients**

27. Which of the following patient features would prompt you to be alert to vitamin D status?

Please tick as many as required:

Dark skin (Fitzpatrick skin type V-VI) ☐[01]
Depression ☐[02]
Women in general ☐[03]
Past history of bone fractures ☐[04]
Current bone disease ☐[05]
Obesity ☐[06]
People who are housebound or institutionalised ☐[07]
Aged over 65 years ☐[08]
People who wear concealing clothing for cultural or religious reasons ☐[09]
Current skin disease ☐[10]
Fatigue ☐[11]
Very fair skin (Fitzpatrick skin type I-II) ☐[12]
Not sure ☐[13]
Pregnant or breastfeeding women ☐[14]
Muscle aches and weakness ☐[15]
Children (aged under 16 years) ☐[16]
Poor nutrition ☐[17]

28. Please estimate the number of patients, for whom you have made a laboratory investigation for serum vitamin D in the LAST 12 MONTHS?

☐☐☐ **patients**

29. Please estimate the percentage of your serum vitamin D orders in the LAST 12 MONTHS that were requested by patients?

☐☐ **percent**

30. Of the patients for whom you have ordered a serum vitamin D test in the LAST 12 MONTHS, approximately which percentages showed vitamin D insufficiency and deficiency?

insufficiency ☐☐ **percent**

deficiency ☐☐ **percent**

31. In which ways do you manage vitamin D insufficiency?

Please tick as many as required in each column:

	**Summer**	**Winter**
Monthly high dose vitamin D supplements	☐[1]	☐[1]
Advice to receive more natural sunlight	☐[2]	☐[2]
Calcium supplements	☐[3]	☐[3]
Advice to receive more artificial UV light	☐[4]	☐[4]
Nutrition advice	☐[5]	☐[5]
Daily low dose vitamin D supplements	☐[6]	☐[6]

32. In which ways do you manage vitamin D deficiency?

Please tick as many as required in each column:

	Summer	Winter
Calcium supplements	☐1	☐1
Advice to receive more natural sunlight	☐2	☐2
Advice to receive more artificial UV light	☐3	☐3
Daily low dose vitamin D supplements	☐4	☐4
Nutrition advice	☐5	☐5
Monthly high dose vitamin D supplements	☐6	☐6

33. For how many patients have you prescribed high dose vitamin D supplements in the past 12 months?

☐☐☐ **patients**

34. Your comments on vitamin D or this survey are most welcome…

____

____

____

____

____

____

____

Thank you for completing this survey. We appreciate your participation.
