# Supplementary material for: “Prescribing sunshine”: a national, cross-sectional survey of 1,089 New Zealand general practitioners regarding their sun exposure and vitamin D perceptions, and advice provided to patients
Source: BMC Fam Pract. 2012 Aug 17;13:85. doi: 10.1186/1471-2296-13-85 (PMC3460728; doi:10.1186/1471-2296-13-85)
Supplement: Additional file 3 — Survey information sheet provided to all respondents. [file 1471-2296-13-85-S3.pdf]

Additional File 3. Survey information sheet provided to all respondents

Survey Information Sheet – Keep this nearby.

The following definitions are used in the context of this survey:

Fitzpatrick Skin types:

1. Skin type I: Always burns, never tans; sensitive to sun exposure,
2. Skin type II: Burns easily, tans minimally; fair-skinned,
3. Skin type III: Burns moderately, tans gradually to light brown
4. Skin type IV: Burns minimally, tans to moderately brown skin
5. Skin type V: Rarely burns, tans to dark brown skin
6. Skin type VI: Rarely burns, least sensitive; deeply pigmented skin

Peak UV times:

Note that the term "peak UV time" refers to 10am to 3pm
(or 11am to 4pm during daylight savings time).

Serum 25(OH)D / vitamin D levels:

Vitamin D deficiency: below 25 nmol/L
Vitamin D insufficiency: between 25 and 50 nmol/L
Adequate vitamin D: 50 nmol/L or above
Source: Australian New Zealand Bone and Mineral Society, 2005

Sun protection:

Use of long sleeved shirts and pants, a hat, sunglasses, sunscreen and shade.
