# Supplementary material for: “Prescribing sunshine”: a national, cross-sectional survey of 1,089 New Zealand general practitioners regarding their sun exposure and vitamin D perceptions, and advice provided to patients
Source: BMC Fam Pract. 2012 Aug 17;13:85. doi: 10.1186/1471-2296-13-85 (PMC3460728; doi:10.1186/1471-2296-13-85)
Supplement: Additional file 4 — Analysis of other associations. [file 1471-2296-13-85-S4.pdf]

Additional file 4: Analysis of other associations

Table A1. Unadjusted and adjusted ratio of geometric means (RGM) and 95% confidence intervals for association of factors with minutes of unprotected sun exposure (face, hands and arms) considered necessary for vitamin D adequacy in people with high sun sensitivity.

	**Unadjusted**				**Adjusted***			
	RGM	*95% CI*		*p*	*RGM*	*95% CI*		*p*
		Lower	Upper			Lower	Upper	
**Location** *ref. all other*				0.538				
Major metropolitan centre with a medical school	0.98	0.91	1.05		1.01	0.93	1.11	0.745
**Place of medical graduation**								
Ref. NZ/Australia				0.080				0.363
US/UK/Other European	1.03	0.94	1.13		1.02	0.92	1.12	
SE Asian	0.80	0.63	1.01		0.85	0.67	1.08	
South African	0.98	0.81	1.19		0.97	0.80	1.18	
All others	0.80	0.64	0.99		0.84	0.67	1.05	
**Sex** *Ref. male*				0.137				0.697
Female	0.95	0.88	1.02		0.98	0.90	1.07	
**Years as GP** *(per 5 years)*	1.03	1.01	1.04	0.004	1.02	1.00	1.04	0.026
**Latitude Band** of practice								
Ref. 40-41.59°				0.099				0.345
Upper-North (34-36.59°)	0.96	0.86	1.07		0.97	0.87	1.09	
Mid-North (37-39.59°)	1.05	0.94	1.17		1.05	0.93	1.18	
Mid-South (42-44.59°)	1.11	0.98	1.25		1.08	0.95	1.23	
Lower-South (45-47°)	1.06	0.91	1.24		1.08	0.93	1.26	
**Information sources read**								
Ref. none								
CSNZ Position statement	0.88	0.81	0.96	0.005	0.90	0.82	0.99	0.026
WHO / IARC	0.96	0.81	1.14	0.652	1.02	0.86	1.23	0.791
Clinical Practice Guidelines	0.89	0.83	0.95	0.001	0.88	0.82	0.95	0.001
NHMRC	1.02	0.92	1.15	0.670	1.09	0.96	1.23	0.169
Any of the above	0.88	0.82	0.94	<0.001				
**Number of sessions** *Ref. ≥8*				0.215				0.195
1-3	1.11	0.98	1.26		1.12	0.99	1.27	
4-7	1.11	0.98	1.26		1.11	0.98	1.26	
**Skin cancer training course**								
Ref. no course completed				0.732				0.788
Completed course	0.98	0.90	1.08		0.99	0.90	1.09	

* Adjusted for all other variables listed in the table.

Table A2. Unadjusted and adjusted ratios of geometric means (RGM) and 95% confidence intervals for association of factors with minutes of unprotected sun exposure (face, hands and arms) considered necessary for vitamin D adequacy in people with low sun sensitivity.

	**Unadjusted**				**Adjusted***			
	RGM	*95% CI*		*p*	*RGM*	*95% CI*		*p*
		Lower	Upper			Lower	Upper	
**Location** *Ref. all other*				0.336				0.375
Major metropolitan centre with a medical school	1.04	0.96	1.13		1.05	0.95	1.16	
**Place of medical graduation**								
Ref. NZ/Australia				0.011				0.014
US/UK/Other European	0.96	0.86	1.07		0.94	0.84	1.05	
SE Asian	0.81	0.62	1.04		0.80	0.61	1.04	
South African	0.84	0.67	1.05		0.85	0.68	1.06	
All others	0.68	0.53	0.88		0.69	0.53	0.89	
**Sex** *Ref. male*				0.728				0.633
Female	0.99	0.91	1.07		0.98	0.88	1.08	
**Years as GP** *(per 5 years)*	1.00	0.98	1.02	0.895	1.00	0.97	1.02	0.702
**Latitude Band** of practice								
Ref. 40-41.59°				0.292				0.713
Upper-North (34-36.59°)	0.96	0.85	1.09		0.99	0.87	1.12	
Mid-North (37-39.59°)	1.01	0.89	1.14		1.04	0.91	1.19	
Mid-South (42-44.59°)	1.11	0.96	1.28		1.07	0.92	1.24	
Lower-South (45-47°)	1.04	0.87	1.24		1.08	0.90	1.29	
**Information sources read**								
Ref. none								
CSNZ Position statement	0.96	0.87	1.06	0.430	0.95	0.85	1.06	0.353
WHO / IARC	1.16	0.96	1.41	0.121	1.25	1.02	1.54	0.033
Clinical Practice Guidelines	0.91	0.84	0.99	0.034	0.90	0.83	0.99	0.026
NHMRC	1.03	0.91	1.17	0.623	1.06	0.92	1.21	0.446
Any of the above	0.91	0.84	0.99	0.027				
**Number of sessions** *Ref. ≥8*				0.373				0.346
1-3	1.10	0.96	1.27		1.11	0.96	1.28	
4-7	1.06	0.92	1.22		1.07	0.93	1.24	
**Skin cancer training course**								
Ref. no course completed				0.321				0.560
Completed course	0.95	0.85	1.05		0.97	0.86	1.08	

* Adjusted for all other variables listed in the table.

Table A3. Factors associated with ‘quality’ of summer sun protection advice for the general population, unadjusted and adjusted odds ratios (OR) and 95% confidence intervals (CI).

	Unadjusted				Adjusted *			
	OR	*95% CI*		*p*	*OR*	*(95% CI)*		*p*
		Upper	*Lower*			*Upper*	*Lower*	
**Location** *Ref. ‘all other’*				0.550				0.699
Major metropolitan centre with a medical school	0.92	0.71	1.20		0.94	0.67	1.31	
**Place of medical graduation** *Ref. NZ/Australia*				0.071				0.014
US/UK/Other European	1.38	0.95	2.01		1.54	1.04	2.29	
SE Asian	0.61	0.29	1.29		0.51	0.23	1.13	
South African	0.56	0.29	1.09		0.55	0.28	1.09	
All other	0.82	0.38	1.77		0.62	0.28	1.38	
**Sex** *Ref. male*				0.344				0.698
Female	1.14	0.87	1.48		1.07	0.77	1.48	
**Years as a GP** *per 5 years*	0.89	0.83	0.95	<0.001	0.86	0.80	0.93	<0.001
**Latitude band of practice** *Ref. 40-41.59°*				0.950				0.883
Upper-North (34-36.59°)	0.98	0.66	1.44		1.01	0.66	1.56	
Mid-North (37-39.59°)	1.01	0.67	1.52		0.95	0.60	1.49	
Mid-South (42-44.59°)	0.85	0.54	1.34		0.82	0.51	1.32	
Lower-South (45-47°)	0.97	0.55	1.69		0.90	0.50	1.61	
**Information sources read**								
CSNZ position statement	1.27	0.90	1.79	0.167	1.33	0.92	1.92	0.132
WHO / IARC	0.77	0.42	1.41	0.395	0.85	0.44	1.65	0.634
Clinical practice guidelines	1.28	0.98	1.68	0.070	1.33	0.99	1.79	0.061
NHMRC	1.00	0.66	1.52	0.998	0.87	0.55	1.38	0.564
Any of the above	1.38	1.06	1.80	0.016				
**Number of sessions** *Ref. ≥8*				0.542				0.250
1-3	1.15	0.74	1.79		1.13	0.72	1.79	
4-7	1.26	0.82	1.94		1.41	0.89	2.26	
**Skin cancer training course** *Ref. no course completed*				0.605				0.620
Completed course	1.10	0.77	1.56		1.10	0.76	1.60	

* Adjusted for all other variables listed in the table.

Table A4. Factors associated with quality of winter sun protection advice for the general population, unadjusted and adjusted odds ratios (OR) and 95% confidence intervals (CI).

	Unadjusted				Adjusted			
	OR	*95% CI*		*p*	*OR*	*(95% CI)*		*p*
		Upper	*Lower*			*Upper*	*Lower*	
**Location** *Ref. ‘all other’*								
Major metropolitan centre with a medical school	1.02	0.80	1.29	0.884	1.01	0.74	1.36	0.963
**Place of medical graduation** *Ref NZ/Australia*				0.680				0.568
US/UK/Other European	1.10	0.80	1.52		1.13	0.81	1.58	
SE Asian	1.52	0.72	3.24		1.62	0.74	3.54	
South African	1.35	0.70	2.63		1.42	0.72	2.78	
All other	0.88	0.42	1.83		0.87	0.41	1.83	
**Sex** *Ref. male*								
Female	1.14	0.89	1.45	0.294	1.12	0.84	1.50	0.429
**Years as a GP** *per 5 years*	0.98	0.93	1.04	0.571	0.99	0.93	1.06	0.816
**Latitude band of practice** *Ref. 40-41.59°*				0.359				0.417
Upper-North (34-36.59°)	1.23	0.86	1.75		1.19	0.81	1.74	
Mid-North (37-39.59°)	1.03	0.72	1.49		1.01	0.68	1.51	
Mid-South (42-44.59°)	1.00	0.66	1.52		0.94	0.61	1.45	
Lower-South (45-47°)	0.77	0.46	1.27		0.75	0.44	1.26	
**Information sources read**								
CSNZ position statement	0.96	0.71	1.29	0.788	0.94	0.68	1.30	*
WHO / IARC	0.88	0.50	1.56	0.663	0.73	0.39	1.36	*
Clinical practice guidelines	1.16	0.91	1.48	0.224	1.10	0.85	1.44	*
NHMRC	1.41	0.96	2.07	0.082	1.41	0.93	2.15	*
Any of the above	1.13	0.88	1.43	0.338				
**Number of sessions** *Ref. ≥8*				0.750				0.819
1-3	1.03	0.68	1.56		1.06	0.69	1.62	
4-7	0.93	0.62	1.39		0.96	0.63	1.48	
**Skin cancer training course** *Ref. no course completed*								
Completed course	0.91	0.66	1.25	0.560	0.91	0.65	1.26	0.560
